# Fibre supplementation alters the gastrointestinal microbiome, the microbial metabolites and indicators of neurodegeneration in a mouse model of Alzheimer´s disease

**DOI:** 10.1038/s41598-025-20986-8

**Published:** 2025-09-24

**Authors:** Linda F. Böswald, Jasmin Wenderlein, Martin Bachmann, Annette Zeyner, Klaus Neuhaus, Frederike Schäfer, Axel Imhof, Shibojyoti Lahiri, Josephine Gruetzke, Bastian Popper

**Affiliations:** 1 Core Facility Animal Models, Biomedical Center, Medical Faculty, Ludwig-Maximilians- Universität München, Großhaderner Str. 9, DE-82152 Planegg-Martinsried, Germany; 2 German Federal Institute for Risk Assessment, Max-Dohrn-Str. 8-10, 10589 Berlin, Germany; 3 Department of Veterinary Sciences, Faculty of Veterinary Medicine, Institute for Infectious Diseases and Zoonoses, LMU Munich, Veterinärstr. 5, Oberschleißheim, Germany; 4 Institute of Agricultural and Nutritional Sciences, Martin Luther University Halle- Wittenberg, Theodor-Lieser-Str. 11, 06120 Halle (Saale), Germany; 5 Core Facility Microbiome, ZIEL Institute for Food & Health, Technical University of Munich, Weihenstephaner Berg 3, Freising, Germany; 6 Faculty of Medicine, Biomedical Center, Protein Analysis Unit, Ludwig-Maximilians- Universität München, Großhaderner Str. 9, DE-82152 Planegg-Martinsried, Germany

**Keywords:** Inulin, Prebiotic, Microbiota, SCFA, Neurology, 5xFAD mouse, Biochemistry, Diseases, Microbiology, Neurology, Neuroscience

## Abstract

Alzheimer´s disease is a neurodegenerative disease with high global prevalence and no cure available. It is known that the microbiota-gut-brain-axis plays a role in the pathogenesis, but the pathways are not fully understood yet. To elucidate the role of dietary fibre supplementation on this axis in a 5xFAD mouse model of Alzheimer´s disease, a feeding trial with an inulin supplement was conducted. At the start (Basis, *n* = 11) and after 7 weeks with (AD + F; *n* = 15) and without (AD; *n* = 15) supplementation, the mice were sacrificed and the following samples were taken: ingesta for 16 S rRNA sequencing and short-chain fatty acid (SCFA) analysis, and brain tissue for amyloid-beta staining and proteome analysis. The microbiota patterns in stomach, small intestine, caecum and colon differed between AD and AD + F. SCFA concentrations were significantly higher in group AD + F as compared to AD and Basis. In the AD mice, plaque load was significantly increased as compared to Basis, while a reduction in AD + F as compared to AD was observed. The brain proteome also differed between AD + F and AD, indicating a beneficial effect of the inulin supplementation, possibly mediated in part by microbial acetate. Since prebiotic substances like inulin are also part of human diets, this should be investigated further in the translational context.

**Supplementary Information:**

The online version contains supplementary material available at 10.1038/s41598-025-20986-8.

## Introduction

Alzheimer´s disease is a progressive neurodegenerative disease causing a specific form of dementia mostly in the elderly. The large global number of affected patients is still increasing and there is no convincing treatment option in broad clinical use^1^.

The pathomechanism of Alzheimer´s disease is explained by the amyloid-beta (Aβ) protein deposition in form of plaques and the extracellular tau tangles. Secondary changes are inflammative responses, oxidative stress and degeneration of nervous tissue^2^. Latest research has shown strong evidence of a connection between the gastrointestinal microbiome and the peripheral and central nervous system via the so-called microbiota-gut-brain-axis^2–7^. A shift in the microbial community may be involved in the neurodegenerative processes. There are several mechanisms by which microbiota can influence the host organism and have neuro-modulating functions, for instance via their metabolites and the vagus nerve^7^. The microbial community can also contribute to systemic inflammation. Short-chain fatty acids (SCFA) as microbial metabolites are of special importance, since a proportion of them enters the bloodstream and can reach the brain, passing through the blood-brain-barrier^8^. In addition, the microbiome is connected with the enteric neuronal system, the integrity of the intestinal barrier^7^ and it plays a role in neurodegenerative disease^9^.

Clinical evidence shows differences in the microbiome patterns of Alzheimer´s patients and healthy controls^10–12^. There is data suggesting an association between the microbiome, the microbial SCFA^13,14^ and the functional and histological characteristics of Alzheimer´s disease in human patients^13^ as well as in rodent models^15–17^. Interventions to alter the intestinal microbiome have been proposed as therapeutic options in human Alzheimer´s patients, aiming at a reduction of Aβ plaques, tau tangles and the resulting neurodegeneration^11,12^.

The 5xFAD transgenic mouse is an extensively studied model for late-onset Alzheimer´s disease, its name standing for the five genetic alterations of *F*amilial *A*lzheimer´s *D*isease. These animals show altered activity and behaviour, progressive cognitive deficits and accumulation of Aβ in the brain^18–22^, mimicking the human disease pathogenesis. In addition, an altered gut microbiome was found in 5xFAD mice^23^. There are some studies in which the gut microbiome was manipulated in 5xFAD mice in order to influence the Alzheimer´s phenotype^24–26^ via the above-mentioned gut-microbiota-brain-axis. Positive effects on cognitive function and brain plaque density were detected for example by prebiotic mannan-oligosaccharide supplementation, or by administration of probiotics or SCFA, respectively^24,26–28^. Considering these findings, microbiome alteration by pre- or probiotics (defined as substrate for microbiota and living gut microbiota, respectively), or their metabolites, seems to be a promising area of research to find preventive or therapeutic options for Alzheimer´s patients. Those dietary interventions could be implemented to slow down the disease progression or alleviate the cognitive decline. To optimize such strategies, it is necessary to understand the connection between the gut microbiome and the central nervous system in more detail and identify the linking signal pathways.

The purpose of the present study was to study the 5xFAD mouse model during the period of disease onset with and without the addition of an inulin supplement. Inulin is a plant storage carbohydrate made up of fructose polymers, serving as a dietary fibre with prebiotic characteristics^29^. We investigated the connection between the intended microbiome alteration and potential effects in the brain, starting at the level of intestinal SCFA and mucous membranes. Analysis of the microbiome from defined sites throughout the gastrointestinal tract was conducted by 16 S rRNA gene amplicon sequencing to characterize the intestinal microbial communities. Two hypotheses were formulated: (a) Dietary fibre supplementation alters the intestinal microbiome and leads to increased fermentation in the hindgut, resulting in higher concentrations of microbial metabolites such as SCFA; (b) Plaque load in the central nervous system will be alleviated by the dietary influence on the gut-microbiome-brain-axis.

## Materials and methods

### Animals

In the study, we used 41 male 5xFAD mice (purchased from Charles River Laboratories GmbH, Sulzfeld, Germany) in accordance with the appropriate European and German animal welfare legislations (5.1–231 5682/LMU/BMC/ project reference number CAM 2023-011). The project was approved by the animal welfare body of the Core Facility Animal Models, BMC, LMU, and all conditions regarding the use of animals are reported in accordance with the ARRIVE guidelines. After delivery from the breeder (Charles River), the animals were housed under specified-pathogen-free conditions in husbandry rooms with defined climate (room temperature 20–22 °C, relative humidity 45–55%, light cycle 12 h light:12 h dark, room air exchange 11x per hour) in individually ventilated cages (HEPA-filtered air flow). The mice were kept in Type II long cages (Tecniplast S.p.A., Buggugiate, Italy) with aspen bedding material (LAS bedding PG3, Altromin Spezialfutter GmbH Co., Lage, Germany), a red corner house (Tecniplast) and enrichment (5 × 5 cm nestlet, Datesand, UK; plastic tunnel). The enrichment items were standard equipment for every mouse cage in the facility at the time so that every cage received the same items. Room air was exchanged 11 times per hour and filtered with HEPA-systems. The hygiene monitoring adhered to the FELASA-14 recommendations (every three months). Two mice were kept per cage. Cages were changed to fresh bedding every two weeks, while daily checks were made to ensure sufficient feed and water availability. Daily feed and water intake were not quantified during the complete study, but no major deviations from the routine observations were noted. These housing conditions were upheld during the period of adaptation to the husbandry until the start of the study and throughout the study.

### Diet and supplement

All animals in the study had *ad libitum* access to the regular diet in the facility at all times. This was the pelleted breeding diet for rats and mice by Altromin (1314P, irradiated, 89.5% dry matter, 23.5% crude protein, 4.0% crude ash, 5.4% crude fat, 4.6% crude fibre, 52.0% N-free extracts). In addition, the AD + F group received an inulin-containing fibre supplement (FiberBites, Clear H_2_O Inc., Westbrook, UK; 1.6 g fibre per piece of FiberBite, mainly from chicory root fibre. Further ingredients according to manufacturer: Sugar, water, pectin, natural flavours, citric acid, sodium citrate, colourants, coconut oil, carnauba wax). Demineralized, filtered water was available at all times.

### Study design

The animals were adapted to the facility and the standard diet for 7 weeks. At the start of the study (t0, age 3 months), 11 randomly selected mice, the Basis group, were sacrificed and dissected (brain, caecum, colon for histology; ingesta of caecum and colon for SCFA analysis). The other mice were randomly allocated to two experimental groups: group AD (*n* = 15) was fed the standard diet without any additional treatment or supplement. Group AD + F (*n* = 15) was fed the standard diet and received the FiberBite supplement for additional free consumption. The FiberBite is a gelatinous feed item (called “gummy” by the manufacturer) with about 1 cm diameter. It was offered separately to the regular diet in the AD + F group. The consumption was monitored qualitatively by regular visual checks to the FiberBites for bite marks.

The mice were weighed weekly. After 7 weeks of the respective feeding regimen (t7, age 4.5 months), the mice were sacrificed by intraperitoneal injection of an overdose of ketamine and xylazine, and dissected. On the days of sacrifice, one mouse was sacrificed and dissected immediately before the next mouse was sacrificed. The sequence of the mice (i.e. distribution of groups) was randomized so as not to have a pattern throughout the day.

Cardiac blood samples were taken and blood glucose was measured (FreeStyle Lite glucometer, Abbott, Canada). The glucose measurement was repeated at least two times and the arithmetic mean of the values was calculated. Samples of gastrointestinal content at defined sites were taken for 16 S rRNA sequencing or SCFA analysis, respectively. The brain was extracted *in toto* and processed as described below.

### DNA extraction and 16 S rRNA gene amplicon sequencing

Ingesta samples were obtained at t7 from stomach (STO), ileum (ILE), caecum (CAE), and colon (COL) from 15 mice of the AD and AD + F group, respectively, as described in detail previously^30^ and stored in 600 µl DNA stool stabilizer (Invitek Molecular GmbH, Berlin, Germany) at -20 °C until further use.

To extract DNA, samples were thawed on ice and a protocol was conducted as described in detail previously (“modification of Godon protocol”^31,32^. Negative controls were extracted along the samples together with a mock community as positive controls (D6300; Zymo Research Europe GmbH, Freiburg, Germany). The library preparation targeting the V3/V4-region of the 16S rRNA gene amplicon was conducted using a two-step PCR with the primers 341F-ovh (5’-CCTACGGGNGGCWGCAG-3’) and 785r-ovh (5’-GACTACHVGGGTATCTAATCC-3’) as previously reported^32^. Sequencing of the generated amplicons was conducted on an Illumina MiSeq system (Illumina Inc., San Diego, CA, USA) platform conducted in a paired-end mode (PE300; using reads of 275 each) with the v3 reagent kit according to the manufacturer’s instructions.

### Short-chain fatty acid analysis

Samples of the intestinal content from the caecum and the colon were used for SCFA analysis (for each site: *n* = 11 at Basis, *n* = 15 for group AD and *n* = 15 for group AD + F). The samples were stored at -80 °C until analysis. The concentrations of acetic acid, propionic acid, n- and iso-butyric acid, n- and iso-valeric acid, and n-caproic acid in caecum or colon digesta, respecticely, were determined by gas chromatography (GC) using a Shimadzu GC2010 (Shimadzu Corp., Kyoto, Japan) fitted with a flame ionization detector. The analytes were separated on an SGE BP21 column (30 m × 0.53 mm × 0.5 μm) (Trajan Scientific and Medical, Ringwood, Australia). A sample weight of 0.02 to 0.47 g was diluted to a 1.8 to 4.1-fold with deionised water. The individual dilution was recorded. The diluted samples were centrifuged for 5 min at 4,000 rpm. An internal standard solution (1.5 g 4-methyl-valeric acid/100 mL formic acid, 80%) was added to the supernatant and the samples were centrifuged for another 5 min at 4,000 rpm. Next, 0.5 µL of the supernatant was injected on-column. The following GC settings were used: 180 °C injection temperature, a constant pressure of 22.7 kPa (i.e., 29.7 cm/s linear velocity and 3.64 mL/min column flow), 85 °C initial oven temperature, raised up by 8 °C/min to 200 °C and held for 6 min, and 200 °C detection temperature. Helium was used as carrier and make-up gas. A 6-point external standard calibration was performed to calculate target SCFA concentrations (quality control data in Supplementary Table T1).

### Histology

The brain was removed *in toto*, fixed in cryo-medium (Tissue Tek O.C.T. Compound, Sakura) in cryomolds and stored at -80 °C. Sagittal brain sections of 5 μm thickness were cut using a Thermo fisher cryotome (Model HM 355 S; Thermo fisher, Life Technologies GmbH, Darmstadt, Germany) and stained with haematoxylin-eosin. Immunohistochemical methods were performed as follows: The sections were blocked with 3% BSA / PBS Tween for 30 min at room temperature. The primary antibody Beta Amyloid Polyclonal Antibody (1:600 dilution, Invitrogen #715800) was incubated overnight at 4 ℃. The next day, a SignalStain^®^ Boost IHC Detection Reagent (HRP, Rabbit) (Cell Signal #8114) was applied undiluted for 1 h at room temperature, followed by a Liquid DAB + Substrate Chromogen System (DAKO #K3467) for the final staining procedure. The samples were counterstained with haematoxylin and finally mounted by using ROTI^®^Histokitt (Roth #6638.1). For negative controls, the primary antibodies were either omitted or replaced by non-immune serum. Images were acquired using an Axiovert 5 microscope connected to an Axiocam 205 camera system (Zeiss, Germany).

### MALDI-imaging mass spectrometry

Five cryo-fixed brains of each experimental group were chosen randomly. Tissues were sectioned on a Cryostar NX50 (Leica Biosystems) into 12 μm coronal sections and thaw-mounted onto indium-tin-oxide (ITO) Slides (Bruker Daltonics GmbH) coated with polyL-Lysine (1:1 water, 0.1% NP40). Sections were stored at -80 °C in a sealed container. The sections were washed according to a pre-established protocol to remove interfering salts and lipids^1^. Briefly, proteins were precipitated in situ by 30 s incubations in 70% ethanol (EtOH) and 100% EtOH. Interferants were removed by 3 min incubation in modified Carnoy’s fluid (60% EtOH, 30% acetic acid, 10% chloroform (v/v/v)) followed by 1 min incubation in 0.1% trifluoroacetic acid (TFA) to acidify the samples. The sections were dried in vacuum. To facilitate Aβ ionization from the tissue, the sections were subjected to formic acid (FA) vapour according to an adapted version of a pre-established protocol^33^. Accordingly, an incubation chamber was heated to 60 °C. On a tissue paper, 3 mL FA were added to a petri dish and incubated for 8 min within the chamber to allow vapour generation. The slides were placed inside the chamber and incubated within the FA vapour for 6 min. Subsequently, the sections were dried in the vacuum chamber. After drying, sections were sprayed with matrix (10 mg/mL sinapic acid (SA) in 50% acetonitrile (ACN); 0.1% TFA) using a HTX TM Sprayer (HTX Imaging, HTX Technologies) using the manufacturers method. Aβ measurements were performed on a rapifleX MALDI tissuetyper MALDI-TOF/TOF mass spectrometer (Bruker Daltonics GmbH). Samples were measured within a mass range of 2000–6000 Da and a spatial resolution of 50 μm using positive reflector mode. A mix of synthetic Aβ peptide species [kindly provided by Prof. Dr. Gerhard Rammes] was spotted at different positions onto the same slide for calibration prior to measurement and as internal standard for peptide identification. The haematoxylin-eosin stained images were co-registered to the software IMS data to enable histological correlation. Aβ peptides were identified by matching with the synthetic Aβ mixture as well as theoretical masses. A list of all potential Aβ peptide masses and intensities were exported for quantification. Intensities of all Aβ peptides were normalized to the internal standard.

### LC-MS/MS

Three brains per group were chosen randomly to be sectioned on a Cryostar NX50 (Leica Biosystems) into 50 μm sections and mounted onto metal frame PET slides (Leica Biosystems). Brain regions were visualized using haematoxlin. In brief, tissue sections were incubated in haematoxylin (Roth #T865.1) for 4 min, followed by blueing under running tap water for 2 min and an ethanol wash for 30 s. A Leica LMD 7000 with a 10x objective was used for LCM. The laser setup was configured as follows: Pulse frequency − 120, maximum pulse energy − 50, aperture − 17, Speed − 10, Head current − 100%, Offset − 65. For every biological replicate, the thalamus and hippocampal areas of dentate gyrus cell layer (G) and *cornu ammonis* pyramidal layer (CA) were manually selected and pooled over three technical replicated. For proteome extraction, the PreOmics iST Kit was used following the manufacturers guide for mammalian tissues (PreOmics GmbH). For effective tissue lysis, the sections were manually homogenized using Micro-homogenizers PP (Carl Roth GmbH + Co KG) after the lysis buffer had been added. Protein digestion was achieved by addition of trypsin/LysC digestion solution and incubation for 3 h at 37 °C. Resulting peptides were desalted, purified, and dried in a vacuum concentrator, followed by resuspension in 12 µL loading buffer for LC-MS/MS measurement.

The LC-MS/MS analysis was carried out on a timsTOF pro (Bruker Daltonics) coupled to a Dionex Ultimate 3000 nanoLC system (Thermo Fisher Scientific). Prior to sample analysis, the instrument was calibrated using a sodium formate solution for mass calibration and Bruker tune mix for mobility calibration. For reversed phase HPLC separation of peptides, 5 µL of the solution were loaded onto a 15 cm analytical column (Bruker PepSep C18 15 cm x 75 μm, 1.5 μm pore size) and separated applying a linear gradient from 3% ACN to 80% ACN over 50 min. Eluting peptides were ionized via Captive Spray ion source (CSI) and analysed in data-independent acquisition mode coupled to parallel accumulation-serial fragmentation (DIA-PASEF) mode. High sensitivity mode was enabled. MS data acquisition was performed in the range from 100 to 1700 m/z with an ion mobility range from 0.6 1/K0–1.6 1/K0. The TIMS analyser was operated in a 100% duty cycle with 100 ms accumulation and ramp times each and a total cycle time of 1.17 s. To monitor instrument performance, quality control samples (50 ng HeLa standard) were included in regular intervals throughout the run (Supplementary Table T2).

Protein identification was carried out using DIA-NN v1.9^34^. Default DIA-NN conditions were used; allowance of missed cleavages was set to 2. For the searches the reviewed mouse canonical protein database from UniProt was used; minimum peptide length was set to 5. DIA-NN output was processed using the R package MS-DAP^35^. Only proteins present in 75% of samples per group were considered. Samples were normalized by “vsn” and “modebetween” and differential expression analysis (DEA) was carried out based on DEqMS algorithm. A list of inflammatory response proteins was generated by filtering the total mouse proteome for the Gene Ontology (GO) term “Inflammatory response” from Uniprot. Volcanoplots were generated by plotting fold change enrichments of the respective proteins between groups using R.

### Statistics

For statistical analysis, GraphPad Prism^®^ v5.04 was used (Graphpad Software, San Diego, CA, USA). The level of statistical significance was set to *α* = 0.05. To compare two groups, unpaired t-tests with Welch´s correction were performed. If all three experimental groups were compared, a one-way analysis of variance (ANOVA) was chosen.

### Analysis of 16 S rRNA amplicon data

Samples of 15 animals in the AD group and 15 animals in the AD + F group were analysed for 16 S rRNA amplicon data. Paired-end reads generated by the Illumina MiSeq (Illumina Inc.) were quality-controlled using fastp v0.23.2^36^, which is included in the AQAMIS pipeline that was executed with the -no assembly mode^37^. Thereafter, fastq files were re-multiplexed using a PEARL script provided on the IMNGS website and processed using IMNGS^38^, an UPARSE-based pipeline^39^, with the following parameters (barcode mismatches: 1; quality score for trimming: 20; minimum ID alignment during merging: 90%; trimming at the forward and reverse side: 20; minimum relative abundance cutoff: 0.25%). Operational taxonomic units (OTUs) were generated by USEARCH v11.0^40^ with an identity of 97% and zero-radius operational taxonomis units (zOTU) by UNOISE2^41^. Non-16 S rRNA sequences were removed using SortMeRNA v4.2^42^ and taxonomic classification was conducted with SINA v1.6.1 using the taxonomy of SILVA release 138^43^. The taxonomy was refined to contain taxa by sequence comparison using EzBioCloud^44^ and a comparison with the LPSN database according to the International Code of Nomenclature of Prokaryotes^45^. The final OTU and zOTU tables for all samples are provided as Supplementary Tables T3 and T4, respectively. In total, 2.7 × 10^6^ sequences with a mean of 21,311 (SD 7787) sequences per sample including negative and control samples were available. All negative controls displayed below 55 reads in total, while all bacterial genera present in the mock community were detected (i.e. *Bacillus subtilis*,* Listeria monocytogenes*,* Staphylococcus aureus*,* Enterococcus faecalis*,* Lactobacillus fermentum*,* Salmonella enterica*,* Escherichia coli*, and *Pseudomonas aeruginosa)*.

Downstream analysis was conducted with Rhea, a modular R pipeline for microbial profiling^46^ (https://github.com/Lagkouvardos/Rhea; accessed on December 21, 2024). The parameter α-diversity was analysed using OTUs using effective richness^47^, while all further analyses were conducted using zOTUs. The parameter β-diversity was calculated via generalized UniFrac distances^48^ using a neighbour joining tree generated with RapidNJ^49^. Significant differences of taxa and zOTUs between experimental groups were calculated using four statistical tests: ALDEx2^50^, ANCOM-BC^51^, Wilcoxon Rank-Sum test^52^, and a linear model^53^, which in combination reveal the most robust results^54,55^. For ALDEx2 v1.34.0 and ANCOMBC2 v2.4.0 count data were analysed with default parameters. The Wilcoxon Rank-Sum test was performed using the stats package v4.1.1 on the normalized relative abundances and the linear model was fitted on TSS normalized counts and log-transformed abundances using lm function of the stats package v4.1.1. The scripts used in this process can be found on github (https://gitlab.com/bfr_bioinformatics/rbvd-study). The *p*-values were corrected using the Benjamini-Hochberg^56^ correction and assumed as significant when below 0.05. Correlations were calculated using Rhea with an adaptation to use the Spearman correlation. Figures were created with R using the functions dplyr v1.1.4, ggplot2 v3.5.1, gridExtra v2.3, readr v2.1.5, reshape2 v1.4.4, tibble v3.2.1, tidyr v1.3.1 and refined using Adobe Illustrator v29.5.1 (Adobe Inc., San José, CA, USA).

## Results

### Energy metabolism

**Fig. 1:**
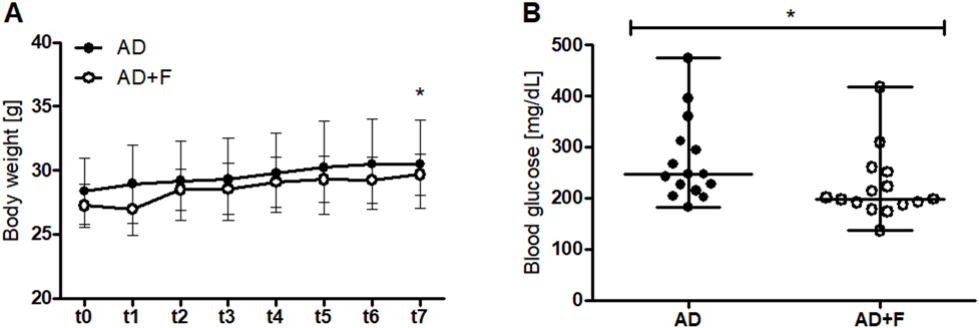
(**A**) Body weight development of the groups AD and AD + F. (**B**) Blood glucose level at t7 of the groups AD and AD + F (*p* < 0.05; dots represent data points; horizontal line marks the median and the whiskers indicate the range).

While there was no significant difference at t0 (*p* = 0.23), the body weight (BW) at t7 differed significantly between the groups AD and AD + F (AD: 30.47 ± 3.46 g; AD + F: 29.67 ± 1.62 g: *p* < 0.01; Fig. 1A). The mice in group AD + F had significantly lower blood glucose levels at t7 than the AD mice (AD: 273.4 ± 81.27 mg/dL; AD + F: 222.1 ± 67.69 mg/dL; *p* < 0.05; Fig. 1B).

### Short-chain fatty acids

There were significant differences between the SCFA concentrations in the ingesta samples of the caecum and colon of Basis, AD and AD + F mice.

**Fig. 2:**
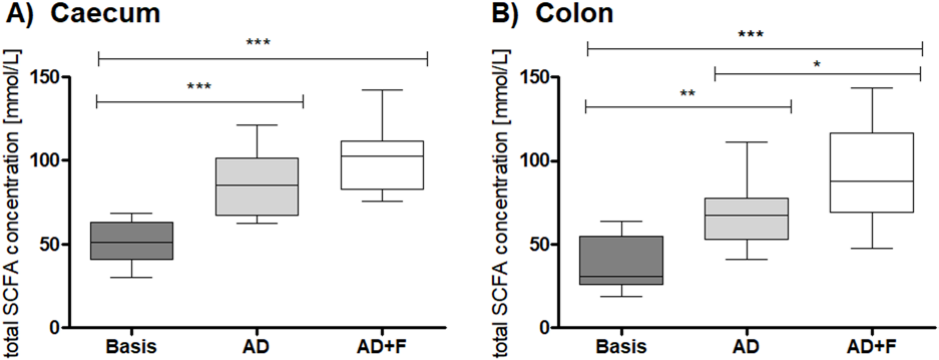
The total SCFA content in (**A**) caecum and (**B**) colon content. Significance indicated by asterisks (***: *p* < 0.001; **: *p* < 0.01; *: *p* < 0.05; boxes represent the middle 50% quartiles separated by the median; whiskers indicate the range).

In the caecum content, the total SCFA concentration was highest in AD + F (101.4 ± 20.7 mmol/L), with intermediate values in group AD (85.8 ± 17.7 mmol/L) and significantly lowest values in the Basis group (52.4 ± 12.7 mmol/L; *p* < 0.0001; Fig. 2A). The concentration of acetic acid, n-butyric acid and propionic acid was significantly lowest in the Basis group and significantly highest in the AD + F group (Supplementary Table T5). The concentration of i- and n-valeric acid was significantly higher in the AD group than both Basis and AD + F. There was a significant difference in the concentration of i-butyric acid between AD + F and AD with AD having higher concentrations, but not compared to Basis.

In the colon content, the concentration of total SCFA differed significantly between all three groups (Fig. 2B; Basis: 37.2 ± 15.7 mmol/L; AD: 66.1 ± 18.1 mmol/L; AD + F: 93.1 ± 30.6 mmol/L; *p* < 0.0001). There were no significant differences in the concentration of i-butyric acid, i- and n-valeric acid between the three groups (see Supplementary Table T5). Acetic acid, n-butyric acid and propionic acid were significantly higher in the AD + F group with lowest values in the Basis group. The pattern of SCFA was not systematically different between the three groups (Supplementary Figure S1).

### 16 S rRNA gene amplicon sequencing of Gastrointestinal samples

**Fig. 3:**
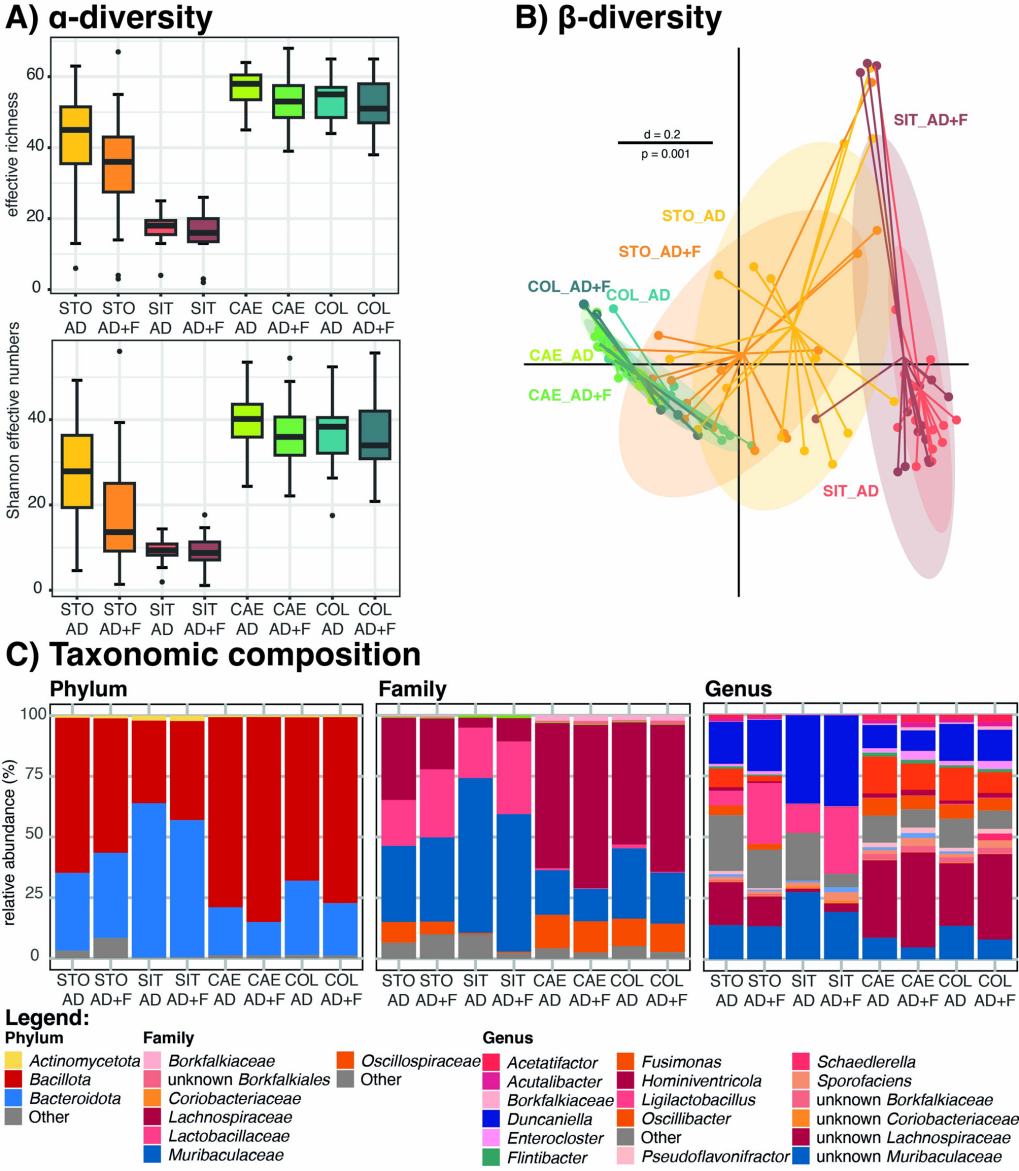
(**A**) α-diversity displayed as effective richness and Shannon effective numbers between different gastrointestinal sites (i.e., STO, stomach; SIT, small intestine; CAE, caecum; COL, colon) and experimental groups; (**B**) β-diversity compared between microbial profiles of gastrointestinal sites and experimental groups displayed as multi-dimensional-scaling plot; the bar indicates percent difference (d), based on generalized UniFrac; *p-*value determination was carried out with PERMANOVA; (**C**) Composition of taxa on phylum, family, and genus level between gastrointestinal sites and experimental groups and depicting results from 16 S rRNA gene amplicon sequencing.

When comparing the bacterial richness of all gastrointestinal regions, slightly lowered values were found in the AD + F group as compared to AD (Fig. 3A). A distinct separation of microbial profiles from small intestine and large intestine was observed, with samples from stomach clustering in between (Fig. 3B). The gastrointestinal site explained 15% of variation among bacterial communities (BC) of the individuals, while the experimental group explained only 3%. The taxonomic composition of all gastrointestinal sites and between the two experimental groups is displayed in Fig. 3C.

**Fig. 4:**
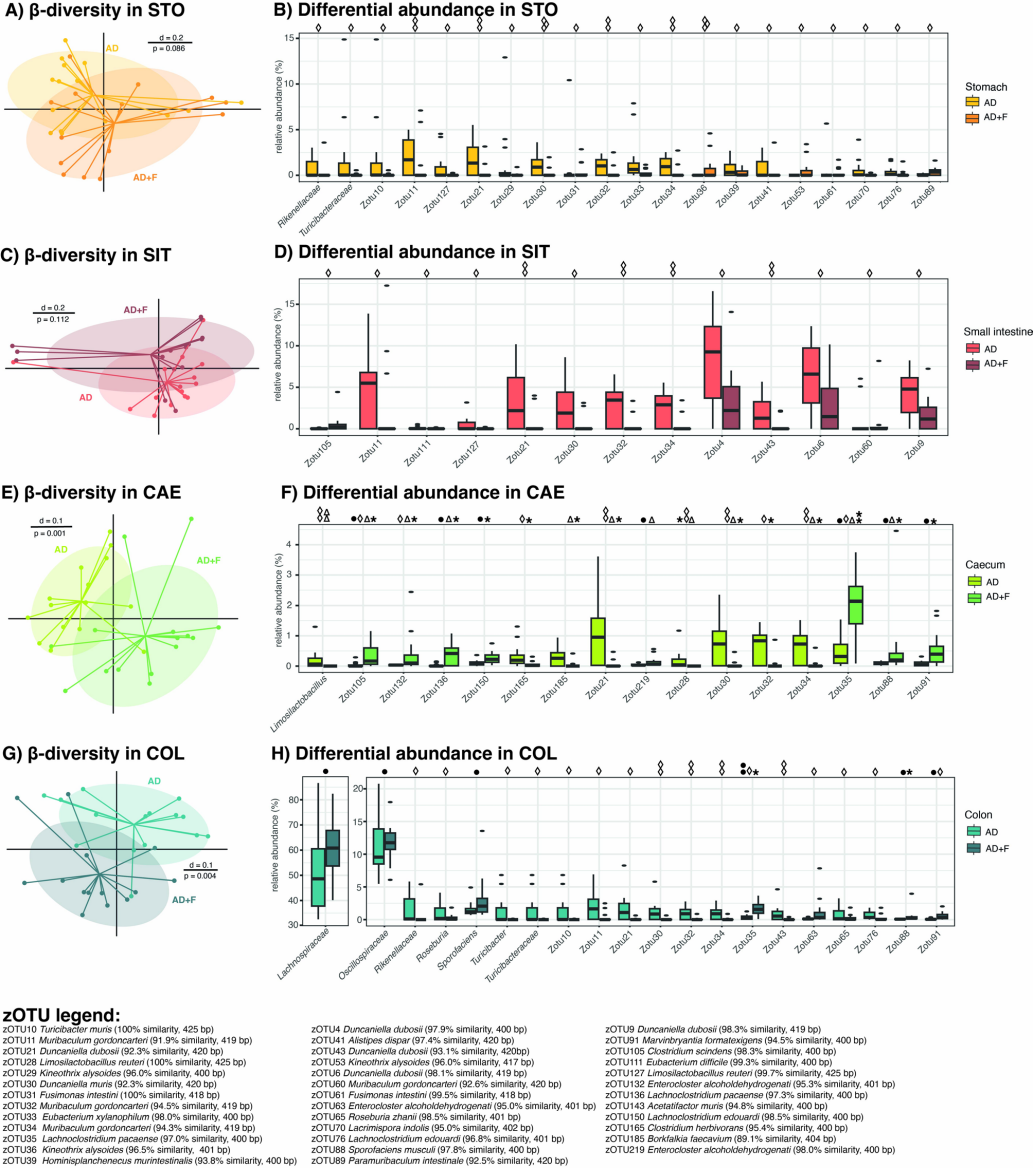
(**A**) β-diversity compared between microbial profiles in the stomach (STO) between experimental groups displayed as multi-dimensional-scaling plot, the bar indicates percent difference (d), based on generalized UniFrac; p value determination was carried out with PERMANOVA; (**B**) Differential abundance in STO between experimental groups calculated with ALDEx2 (●), ANCOM-BC (◊), Wilcoxon Rank-Sum test (*), and a linear model (Δ), displaying the 20 taxa with the highest abundance and significant differences between experimental groups (boxplots display mean, upper and lower percentile in the box and standard deviation as whiskers, and outliers as dots); (**C**) β-diversity compared between microbial profiles in the small intestine (SIT), depicted as in A); (**D**) Differential abundance in SIT between experimental groups, as in B); (**E**) β-diversity compared between microbial profiles in the caecum (CAE), depicted as in A); (**F**) Differential abundance in CAE between experimental groups displaying only taxa that are significant in at least two different statistical tests, as in B); (**G**) β-diversity compared between microbial profiles in the colon (COL), depicted as in A); (**H**) Differential abundance in COL between experimental groups displaying the 20 highest abundant taxa with significant differences between experimental groups, as in (**B**). Significance is indicated by symbols above the graphs (i.e., ALDEx2 (●), ANCOM-BC (◊), Wilcoxon Rank-Sum test (*), and a linear model (Δ)) for pairwise comparison, by the example of the Wilcoxon Rank-Sum test, * *p* ≤ 0.05, ** *p* ≤ 0.01, *** *p* ≤ 0.001. The zOTU legend gives the closest known relative to the respective amplicon, similarity depicts the genetic relation (similarity values > 97% mean the same species is likely, values between 90 and 95% allow for assignment on genus level, values < 90% on family level).

In the samples obtained from stomach and small intestine, microbial profiles displayed no significant difference between the BC of AD and AD + F (Fig. 4A and C). Nevertheless, some taxa and zOTUs differed significantly between these two groups when tested with ANCOM-BC2 (Fig. 4B and D). In the stomach, most significant zOTUs were of higher abundance in the BC of AD, while only zOTU36 and zOTU53, two unknown species of the family *Lachnospiraceae* were of higher abundance in the BC of AD + F (Fig. 4B). In the caecum samples, the microbial profiles differed significantly between the two experimental groups (Fig. 4E) and various taxa and zOTUs were found differing significantly between the two experimental groups´ BCs in at least two of the statistical tests conducted on this dataset (Fig. 4F). Among these, two zOTUs (zOTU35 *Lachnoclostridium pacaensae*, 97.0% similarity, and zOTU105 *Clostridium scindens*, 98.3% similarity) with a higher abundance in AD + F displayed a significant difference in all four tests. It is noteworthy that unknown members of the *Muribaculaceae* family were found to be significantly higher in AD in at least two tests (zOTU21, zTOU30, zOTU32, and zOTU34), while most unknown members of the *Lachnospiraceae* family, *Hominiventricola* spp., and *Enterocloster* spp., were of higher abundance in AD + F BCs. Further taxa that displayed significant differences in their relative abundance between AD and AD + F are displayed in Supplementary Table T6. In the colon samples, a significant difference in the microbial profiles of the two experimental groups BCs (Fig. 4G) was observed. However, only few zOTUs displayed significant differences among multiple statistical tests (e.g. zOTU35 *Lachnoclostridium pacaensae*, 97.0% similarity, zOTU88 *Sporofaciens musculi*, 97.8% similarity, and zOTU91 *Marvinybyantia formatexigens*, 94.5% similarity), while the general composition on family level displayed significant differences in the families *Lachnospiraceae* and *Oscillospiraceae* (Fig. 4H). Considering the significant differences between the four sampling sites, some observations on unknown members of the *Muribaculaceae* family were made throughout all gastrointestinal regions (e.g., zOTU11, zOTU21, zOTU30, zOTU32, zOTU34, and all unknown members of the *Muribaculaceae* family; Fig. 4B, D, F, H). In the hindgut (i.e., CAE and COL), significant results for zOTU35 (*Lachnoclostridium pacaensae*, 97.0% similarity), zOTU88 (*Sporofaciens musculi*, 97.8% similarity), and zOTU91 (*Marvinybyantia formatexigens*, 94.5% similarity) were observed to be significantly different with more than one statistical test were observed in both regions. In addition, significant differences were found with only one test for zOTU63 (*Enterocloster alcoholdehydrogenati*) and zOTU143 (*Acetatifactor muris)*; Fig. 4F and H).

### Histology

Immunohistochemical analysis of the brain sections showed an accumulation of plaques in the hippocampus formation, predominantly in the subiculum region in all three regions. In addition, plaques could be detected in all three groups in the cortex and thalamus regions of the brain slices. Supplementary Figure S2 shows exemplary images of the immunohistochemical brain sections.

### Plaque detection

**Fig. 5:**
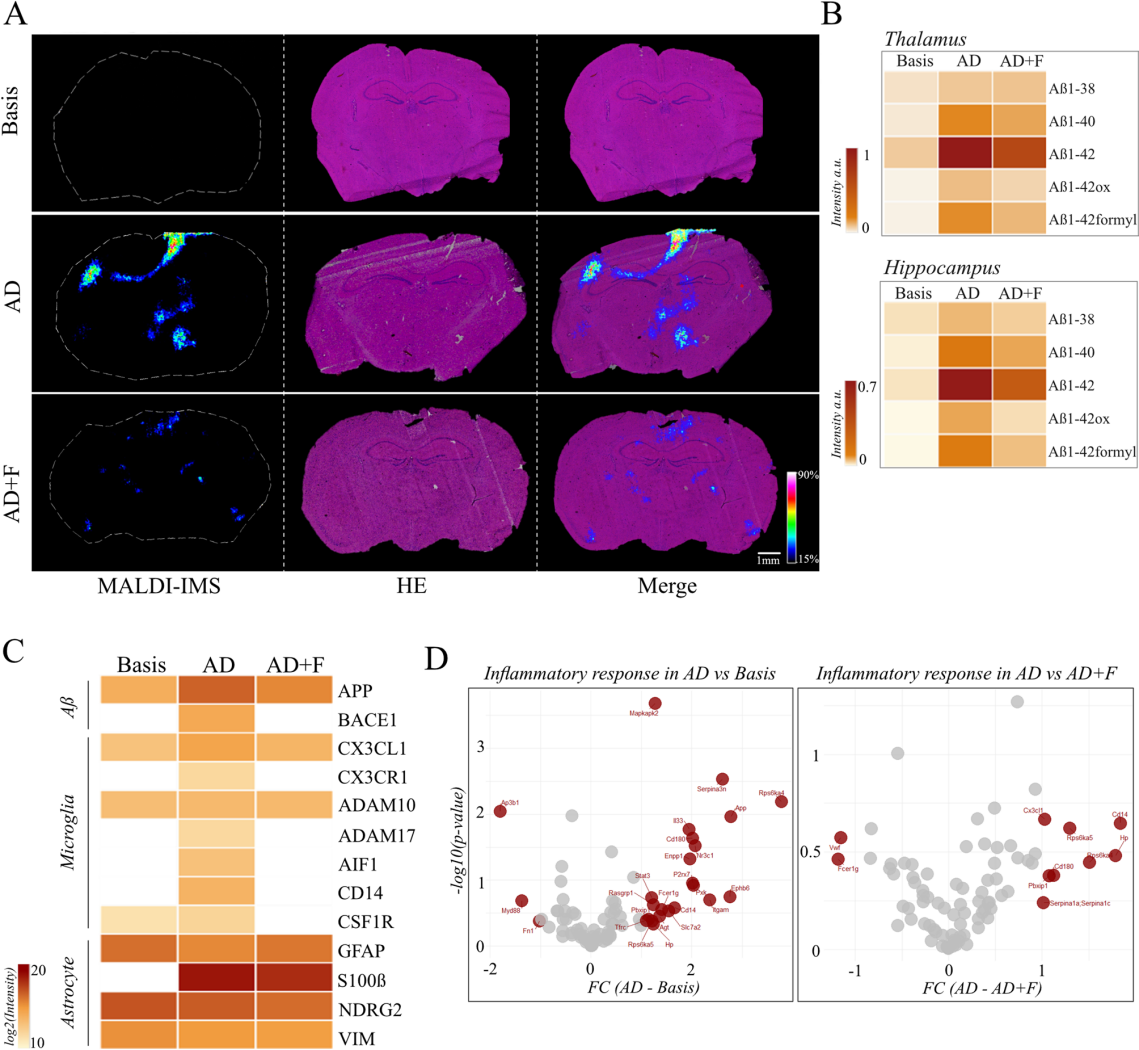
Detection of Aβ species in the mouse brain. (**A**) Representative distribution of m/z 4515.4, identified as hAβ1–42. Presence and abundance are indicated by coloured signal. (**B**) Quantification of Aβ species in thalamus and hippocampus of Basis, AD and AD + F. Displayed are normalized in intensities [a.u.]. (**C**) Protein markers for Aβ production, microglia and astrocytes in thalamus. Quantification by LCM LC-MS/MS. Intensity values are displayed as log2 across Basis, AD and AD + F groups. (**D**) Enrichment analysis of inflammatory response proteins in AD compared to basis group (left) and AD compared to AD + F (right). Significantly enriched proteins are indicated in red, non-significant proteins in grey. Statistical test performed by DeqMS. Statistical significance at *p* < 0.05. Enrichment was defined as log2FC > 1.

Spatial distribution of amyloid depositions was mapped in situ using a specialized protocol for MALDI-Mass Spectrometry Imaging. Treatment with formic acid enabled extraction of amyloid peptides directly from the tissue while degrading other interfering proteins. Consequently, multiple co-localizing Aβ species were detected consistently across animals. They displayed the expected localized distributions of senile plaques across the tissue, being mainly present in thalamus, hippocampus and cortex (Fig. 5A). Most abundant were m/z 4515.4 and m/z 4331.4, matching the theoretical masses of hAβ1–42 (m/z 4514.07) and hAβ1–40 (m/z 4329.86), respectively, as well as the internal standard (Fig. 5A). Additional masses at m/z 4134.9, m/z 4534.4 and m/z 4543.4 were assigned to theoretical masses of hAβ1–38, hAβ1-42ox and hAβ1-42formyl, respectively. Due to lack of inclusion, they could not be further verified by the internal standard mix. Quantification of amyloid peptides in thalamus and hippocampus revealed highest abundance of hAβ1–40, hAβ1–42, hAβ1-42ox and hAβ1-42formyl in AD mice with a marked reduction in AD + F (Fig. 5B). hAβ1–38 levels followed the same trend in hippocampus but showed no difference between AD and AD + F in thalamus (Fig. 5B). All detected peptides showed minor or no presence in the basis group (Fig. 5B).

### Proteome analysis

The majority of analysed proteins in the brain regions were present in all three experimental groups, however, region-specific differences could be found (Supplementary Figure S3). As amyloid deposition and treatment-associated reduction was most prominent in thalamus, the region was isolated using laser capture microdissection (LCM) and the proteomic landscape characterized.

APP (Amyloid-beta precursor protein) and BACE1 (Beta-Secretase 1), the rate limiting enzyme in Aβ production, were quantified to profile Aβ generation. APP and BACE1 levels were notably increased in thalamus of AD mice in comparison the basis group, potentially a manifestation of disease onset. This effect was ameliorated in AD + F (Fig. 5C), where BACE1 could not be detected.

As glial cells play a crucial role in AD development by directly influencing Aβ production and clearance, but also contributing substantially to overall neuronal health^57^, glial cell presence was investigated. Microglia are commonly profiled by expression of AIF1/Iba1 (Allograft inflammatory factor1), CD14 (Monocyte differentiation antigen CD14), CSF1R (Macrophage colony-stimulating factor 1 receptor) and CX3CR1 (CX3C chemokine receptor 1)^58–60^. The release of membrane-bound CX3CL1 (Fractalkine) by ADAM10 or ADAM17 (disintegrin and metalloprotein domain-containing protein 10/17) and recognition by microglial CX3CR1 induces microglial activation^61^ and was therefore considered as well. Astrocytes were identified by presence of GFAP (Glial fibrillary acidic protein), NDRG2 (Protein NDRG2), VIM (Vimentin) and S100β (Protein S100-B)^62^. AIF1, CX3CR1, ADAM17, CD14 and CSF1R were exclusively detected in AD. CX3CL1 levels are highest in AD, while ADAM10 expression showed no difference between groups (Fig. 5C). The expression of astrocytic markers GFAP, NDRG2 and VIM is comparable across groups with a minor reduction of GFAP in AD mice, while S100β levels were highest is AD with a reduction in AD + F (Fig. 5C).

In line with that, overall inflammatory response proteins were enriched in AD mice upon disease onset (Fig. 5D, left). This effect persisted when comparing AD with AD + F, indicating an attenuation of the inflammatory response in AD + F (Fig. 5D, right).

In addition to the thalamus, hippocampal neuronal populations (DG and CA) were profiled for inflammatory markers (Supplementary figure S4). Less microglial and astrocytic markers were detected there as compared to the thalamus, but the proteins present in DG and CA display a similar trend as in the thalamus (Supplementary figure S4A). The overall inflammatory response proteins display less pronounced enrichments than observed in the thalamus (Supplementary figure S4B).

## Discussion

In the present study, we used 5xFAD mice as a proven transgenic model for AD that has been characterized in detail^18,20,22^. The study was conducted at an age of the mice that is typical for the onset and progression of the AD phenotype, as demonstrated by the development of brain changes in group AD vs. group Basis (i.e., the factor time). Thus, this period of time seemed to be well-suited to test interventions aiming to prevent or at least slow down the disease progression, because irreversible neurodegeneration has not fully developed yet.

One limitation is the use of exclusively male mice. There is evidence for sex differences in the brain pathology and cognitive phenotype of 5xFAD mice^63,64^ as well as in human Alzheimer´s patients^65^. Thus, our findings cannot be equally translated to female mice of the same genetics. There may be differences in the response to the dietary intervention that we cannot speculate upon with the data generated. Targeted studies on female mice are surely warranted to see whether the fibre supplementation can also be beneficial for them and to which extent.

The inulin supplement was consumed without noticeable adverse effects by the mice in group AD + F over the trial period of 7 weeks. Alternative to the use of the additional supplement for voluntary consumption, it would be possible to incorporate fibre into a complete feed or to administer fibre via orogastric gavage. The latter would be more invasive and stressful for the animals and is thus not ideal in terms of experimental design. By the addition of a supplement next to the standard diet, we aimed to mimic the situation where a prebiotic supplement was added to a regular diet without influence on the feed intake of the normal diet. Inulin was selected as main fibre of choice because its prebiotic effect has been shown in many species and trial settings before. As a fermentable fibre, inulin is known to increase the SCFA production^66–68^ and to affect the intestinal microbiome^69^. Pectin was also contained in the supplement and it is expected to have been utilized by the microbiota. In a study in rats, pectin was shown to benefit the intestinal epithelium and increase the caecum SCFA content in comparison to a fibre-free diet^70^.

The significantly lower blood glucose levels in the AD + F mice compared to the AD mice at t7 are likely associated with the fibre supplementation, as known from several species. This is supportive of the voluntary consumption of the fibre supplement by all animals within the AD + F groups. Within the gastrointestinal tract, fibre can slow the uptake of nutrients and thus decrease the postprandial glucose peak^71,72^. In addition, indirect effects on glucose homeostasis can be exerted via microbial fermentation of fibre in the hindgut. The microbial alteration of SCFA and bile acid metabolism can affect the endocrine regulation of insulin secretion^73^. Since there is evidence of a link between glucose dysregulation and AD^74^, it seems important to further investigate glucose control in the 5xFAD model and how fibre supplementation can improve this.

### Short-chain fatty acids

For all measured SCFA, the concentration in the caecum content was higher than that in the colon content. This can be explained by the high fermentative activity of the microbiota in the caecum and a subsequent reduction of the metabolite concentration by both cross-feeding^75^ and intestinal absorption^76^. The pattern of higher and lower concentration in the three experimental groups was consistent in caecum and colon. The mice in group AD + F had the significantly highest overall SCFA concentration, followed by group AD. This is in accordance with literature reports of reduced faecal SCFA levels in mouse models of AD^77^ and studies on human patients^78^, even though the limitation of faecal SCFA analysis in those studies needs to be taken into account. Differentiation between SCFA concentrations in caecum and colon as performed in this study renders a better interpretation of the processes involved.

The acetic acid concentration was significantly higher in the caecum and colon content of the AD + F mice as compared to that of the other two groups. This is consistent with the reports of in vivo fermentation experiments with inulin as substrate showing the production of mainly acetate and to lesser extents butyrate and propionate^79^. A study in horses fed inulin and fructo-oligo-saccharides from Jerusalem artichoke meal also showed increased acetate and n-butyrate concentrations in stomach and colon^80^. It has been shown that acetate has neuroprotective and anti-inflammatory effects that can even attenuate cognitive impairment^81^. Among the microbially derived SCFA, acetate seems to be the major factor for microglia maturation and homeostasis^82^. The exact mode of action is not known, but there is evidence that acetate metabolism contributes to a reduction of inflammatory signalling in vitro^83,84^ and in a rat model^85^. Microglia are essential for brain health and tissue regeneration, and their function is impaired in neurodegenerative disease like AD^86,87^. Considering these links, the observed increase in digesta acetate in the AD + F mice might be associated with the results of the Aβ quantification and beneficially altered brain proteome as compared to the AD mice of the same age. However, we cannot prove this potential explanation with the data from the study.

### 16 S rRNA gene amplicon sequencing of Gastrointestinal samples

The present study investigated the bacterial communities in different parts of the gastrointestinal tract by 16 S rRNA sequencing. In comparison to methods like metagenomics, this can only give an overview of the members of the microbial community with limited information about the functions. However, it is a standard method that is well suited to get an overview and there is a lot of literature to reference to. Further metagenomic analyses are certainly desirable in the future.

It is well known that the microbial community strongly differs throughout the gastrointestinal tract^88–91^. This allows for an integrated view of diet-induced changes in the microbial community. The examination of faecal samples was omitted as they do not represent the microbial community or metabolic pattern present in the gastrointestinal tract^30,91–93^, so that the method employed in this study has clear benefits. It is of special importance given that mice are hindgut fermenting rodents^94^, which depend on prae-caecal enzymatic degradation and absorption of easily digestible nutrients as well as microbial fermentation mainly of fibre in caecum and colon. The digestive physiology leads to different digesta and microbiota profiles in the gastrointestinal regions. Indeed, the influence of the inulin supplementation in this study was strongest in the large intestine and especially in the caecum, also supported by β-diversity results (including adonis) and the higher number of statistical tests agreeing in the significance of results in the differential abundance analysis. The use of multiple statistical tests as proposed for microbiome studies^95,96^, and as conducted here, allows for a more robust biological interpretation when multiple tests indicate significance.

Our results of the α-diversities do not agree with a reduction in molecular strain diversity that has been associated with Alzheimer´s disease in human patients^10,97^ and 5xFAD mice^98^. However, a comparison of α-diversities across studies may be limited, especially when non-effective measures are reported. Nevertheless, changes in the microbial profiles of all gastrointestinal regions tested in this study after fibre supplementation are corroborated by previous studies^99,100^.

Most differences on zOTU level were observed for the families *Muribaculaceae* and *Lachnospiraceae*. Unfortunately, many genera and species of these two families have currently not been described or even cultured, which impairs proper discussion. However, it is known that *Muribaculaceae* are main members of the murine gastrointestinal microbiome and can be divided into three subgroups by the expression of the enzyme glycoside hydrolase, which is either specified for α-glucans, host or plant glycans^101^. Similarly, members of the family *Lachnospiraceae* contain glycoside hydrolases, which enable them to degrade various complex carbohydrates (e.g. pectin, hemicellulose, cellulose, starch, alpha-glucans and xylans)^102^ and produce SCFA as fermentation end products^103^. The increased production of SCFA seems to slow down the pathogenesis of Alzheimer´s disease.

Further research in the yet undescribed members of the family *Muribaculaceae* (zOTU21, zOTU30, zOTU32, zOTU34) found in the AD group is required. If these species are, for instance, capable of host glycan and especially mucus degradation, they might be the central link from microbiota to compositional changes of the intestinal mucus layer, a pathomechanism associated with various pathological processes including Alzheimer´s disease^104^. The difficulties in assigning the findings of microbiome studies to functional processes highlight the importance of continued cultivation of members of the mouse intestinal tract to further characterize them, including their capacity to degrade mucin in vitro^105–107^. 16 S rRNA gene amplicon sequencing is not well suited for making statements much beyond genus level, since functions as they are mostly species-, if not strain-specific^96^. Nevertheless, a comparison of the present data with literature might already shed some light in the pathomechanism linked to Alzheimer´s disease.

Of the molecular strains that displayed a similarity over 97% to known species, some were found enriched in relative abundance in the AD group (i.e., zOTU10 *Turicibacter muris*, 100% similarity, 425 bp; zOTU33 *Eubacterium xylanophilum*, 98.0% similarity, 400 bp; zOTU4 *Duncaniella dubosii*, 97.9% similarity, 400 bp; zOTU41 *Alistipes dispar*, 97.4% similarity, 420 bp; zOTU6 *Duncaniella dubosii*, 98.1% similarity, 419 bp; zOTU9 *Duncaniella dubosii*, 98.3% similarity, 419 bp; zOTU111 *Eubacterium difficile*, 99.3% similarity, 400 bp; zOTU127 *Limosilactobacillus reuteri*, 99.7% similarity, 425 bp). While an increase in relative abundance might be associated to the disease onset or progression, opposing results for these bacteria exist in literature. For example, *Turicibacter* spp. have been described to be related to the pathogenesis of Alzheimer’s disease or not^98,108^. This contradiction might be explained by strain-specific functions in bile acid and lipid metabolism^109^. However, for other genera the picture might be clearer. For instance, the genus *Alistipes* – known for its proinflammatory potential^110^ – has regularly been associated with Alzheimer’s disease, cognitive impairment and mental disorders^10,98,110–114^. In contrast, *Limosilactobacillus reuteri* was found to be more abundant in healthy controls than patients with mild cognitive impairment^115^. A relationship between Alzheimer’s disease and *Eubacterium* spp. or *Duncaniella* spp. has not been described previously to the best of the authors´ knowledge.

The species enriched in the mice of group AD + F might allow for some insight into microbiota possibly counteracting the onset or progression of Alzheimer´s disease. The molecular species were, for example, (i.e., zOTU35 *Lachnoclostridium pacaense* (97.0% similarity, 400 bp), zOTU61 *Fusimonas intestini* (99.5% similarity, 418 bp), zOTU88 *Sporofaciens musculi* (97.8% similarity, 400 bp), zOTU105 *Clostridium scindens* (98.3% similarity, 400 bp), zOTU136 *Lachnoclostridium pacaense* (97.3% similarity, 400 bp), zOTU150 *Lachnoclostridium edouardi* (98.5% similarity, 400 bp), and zOTU219 *Enterocloster alcoholdehydrogenat*i (98.0% similarity, 400 bp). In general, *Enterocloster* spp. exert anti-inflammatory and immunomodulatory effects^116^, hinting at a potentially beneficial effect also in the 5xFAD mouse model. The genus *Lachnoclostridium* is part of the *Lachnospiraceae* family known for its capacity for fibre degradation and production of SCFA^102,103^, possibly providing anti-inflammatory effects. Other taxa (e.g. zOTU35, zOTU88, zOTU105, zOTU136, and zOTU21), which were found significantly enriched in AD + F, no matching species information is available. While they have been identified as closely related to each other, their link to Alzheimer´s disease or it´s prevention in 5xFAD mice has not been described yet.

### Plaque detection

A central part of Alzheimer´s pathogenesis is the formation of amyloid plaques. In our study, formation of these senile plaques was profiled in situ using Imaging Mass Spectrometry, detecting several amyloid fragments. Among these, the extracellular fragment 1–42 is considered the most important in Alzheimer´s disease in humans^117^. Thereby, localized deposition of the amyloid proteins hAβ1–40, hAβ1–42, hAβ1-42ox and hAβ1-42formyl was observed in AD and AD + F, but not the younger basis group (Fig. 5A). This effect of ageing matches the known timepoint of disease onset and Aβ deposition in the 5xFAD mouse model, confirming that our method enables tracking disease progression as well as plaque composition^18,118,119^. Figure 5A shows the pattern of plaque deposition in the AD mice, which might be similar to a subpial, band-like plaque in the neocortical layer of the brain described by Walker^120^ in human brains. Furthermore, immunohistochemical staining revealed the formation of plaques in all three groups, which is in accordance with the results of the proteome analysis. The described modified forms of hAβ1–42 may be a product of sample processing. While hAβ1-42ox can also occur naturally, hAβ1-42formyl does not, and is likely a result of the dietary treatment. All detected Aβ species show reduced abundance in AD + F in comparison to AD, with the exception of hAβ1–38, which remains stable across groups (Fig. 5B). This observation hints at a positive effect of inulin supplementation on the brain. The mechanism behind this may be a reduced Aβ production or increased Aβ clearance. Aβ clearance can be promoted via microglia^121,122^, which in turn are beneficially influenced by acetate. The increase in intestinal acetate production discussed above might improve microglia function and in turn contribute to Aβ clearance. To further elucidate the molecular effect of diet intervention, we performed shotgun proteomics. We found increased levels of both APP and BACE1 in AD mice compared to AD + F, indicating that Aβ production seems to be reduced in AD + F mice. Additionally, AD mice displayed elevated inflammatory markers, indicating increased infiltration of microglia and reactive astrocytes. Increased detection of fractalkine together with ADAM 10/17 and CX3CR1 may indicate microglial activation in AD mice (Fig. 5C). The dietary intervention seemingly dampened the overall inflammatory response.

Hippocampal neuronal populations display less pronounced enrichments of inflammatory markers than thalamus. This may indicate less infiltration of microglia and reactive astrocytes. However, as these are dense neuronal populations, proteomic signatures may be diluted by the substantial neuronal contribution. Nonetheless, we could not find an indication for pronounced inflammation in those neuronal populations.

The increased production of SCFA, particularly acetate, in the AD + F group can be connected to these observations. This SCFA can cross the blood-brain barrier and directly influence brain health. Particularly microglial maturation and activity is positively influenced by either acetate directly or its product butyrate^123–125^. As microglia play a pivotal role in avoiding amyloid plaque formation by both active plaque clearance and by stimulating neurons towards non-amyloidogenic pathway of APP processing^123–125^, this matches our observation of reduced Aβ load as well. However, the identification of exact mechanisms behind these observations is beyond the scope of this study and requires further investigation. The present findings are the basis for future translational studies for a targeted nutritional intervention to beneficially influence the gut-microbiota-brain axis in neurodegenerative disease. The overall goal could be to establish nutritional recommendations or personalized supplementation for human risk patients to prevent or slow down the progression of Alzheimer´s disease.

## Conclusion

The feeding trial had impressive effects on the 5xFAD mice along the microbiota-gut-brain axis. The inulin supplementation lead to changes in the intestinal bacterial communities, which supposedly are of high biological relevance in the prevention of Alzheimer´s disease. Several microbiota, including the genus Lachnoclostridium, are likely associated with the increase of SCFA in caecum and colon content of the AD + F mice. The SCFA can act locally on the mucosal integrity as well as in the central nervous system after absorption into the bloodstream. This might be related to a reduced Aβ load in the brains of AD + F mice and a beneficial alteration of brain proteome. Further research is warranted to identify and characterize the functional pathways.

## Supplementary Information

Below is the link to the electronic supplementary material.

Supplementary Material 1

Supplementary Material 2

Supplementary Material 3

Supplementary Material 4

Supplementary Material 5

Supplementary Material 6

Supplementary Material 7

## Data Availability

Microbiome sequencing data is available in the Sequence Read Archive via https://www.ncbi.nlm.nih.gov/bioproject/PRJNA1276483. Further datasets used and/or analysed during the current study are available from the corresponding author on reasonable request.
