# Supplementary material for: Fibre supplementation alters the gastrointestinal microbiome, the microbial metabolites and indicators of neurodegeneration in a mouse model of Alzheimer´s disease
Source: Sci Rep. 2025 Sep 24;15:32705. doi: 10.1038/s41598-025-20986-8 (PMC12460888; doi:10.1038/s41598-025-20986-8)
Supplement: Supplementary file 1 — Supplementary Material 1 [file 41598_2025_20986_MOESM1_ESM.pdf]

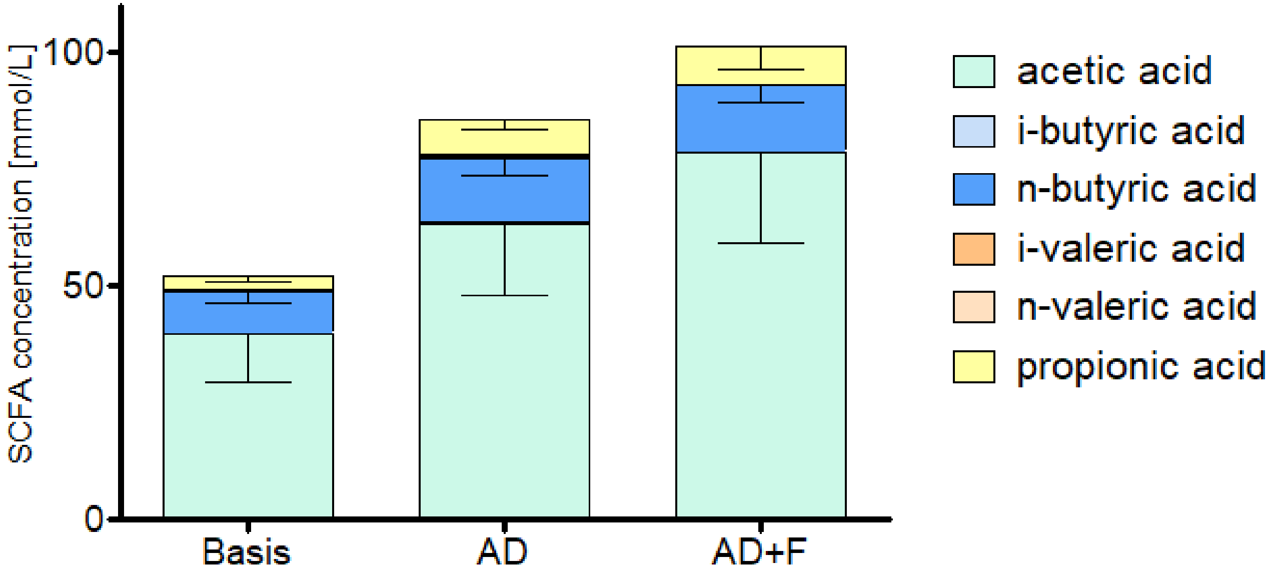


**Supplementary Figure S1.**
Distribution of the individual SCFA in the caecum content.

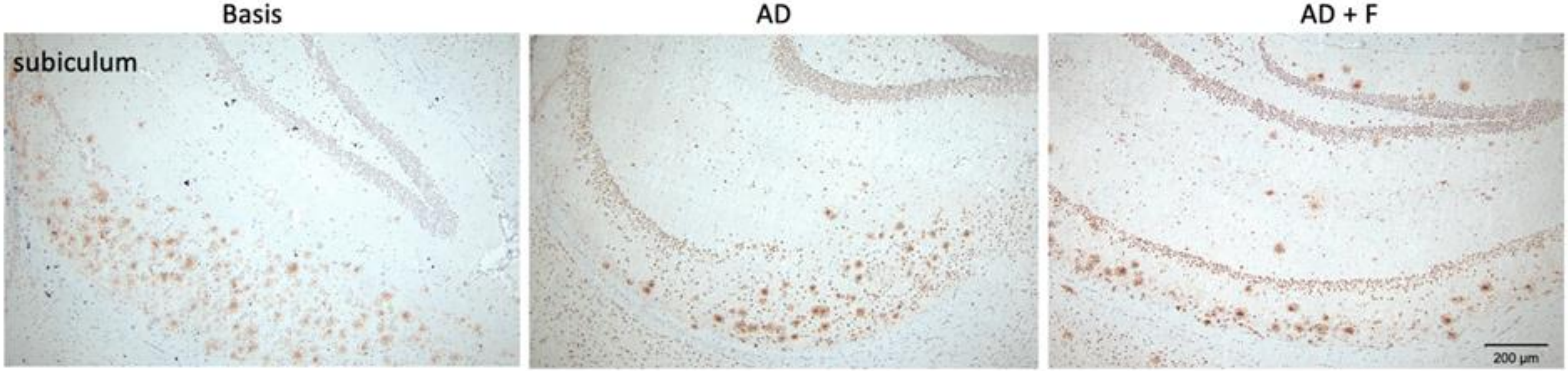


Supplementary Figure S2.
Representative immunohistochemically stained brain sections. Aβ antibody was used to identify plaques in the area of the hippocampal formation in Basis, AD und AD+F mice.

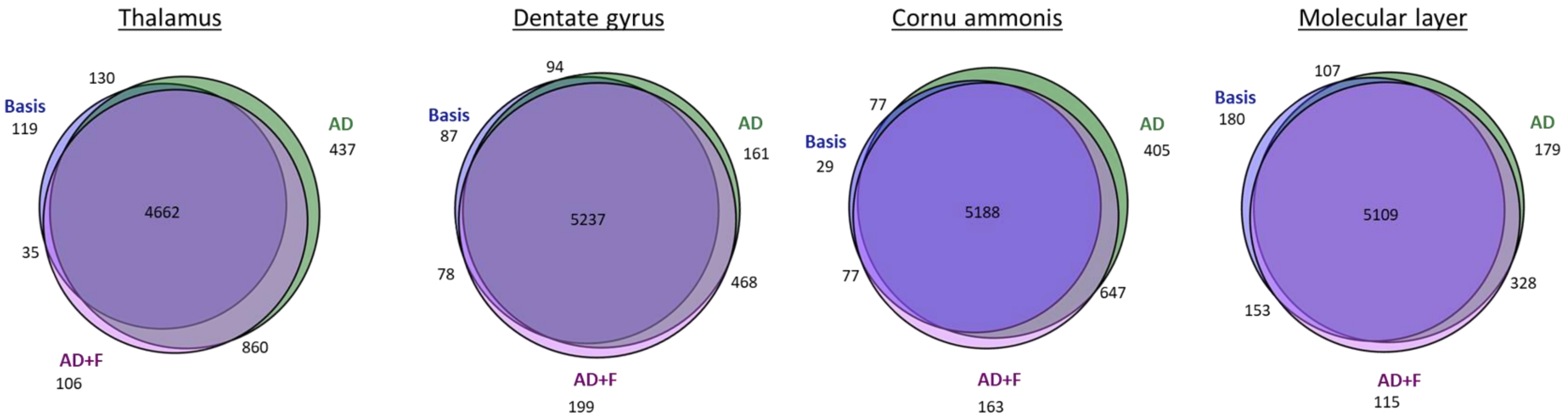


**Supplementary Figure S3.**
Graphical illustration showing the numbers of proteins identified in the brain regions and the overlap or difference in numbers between the experimental groups.

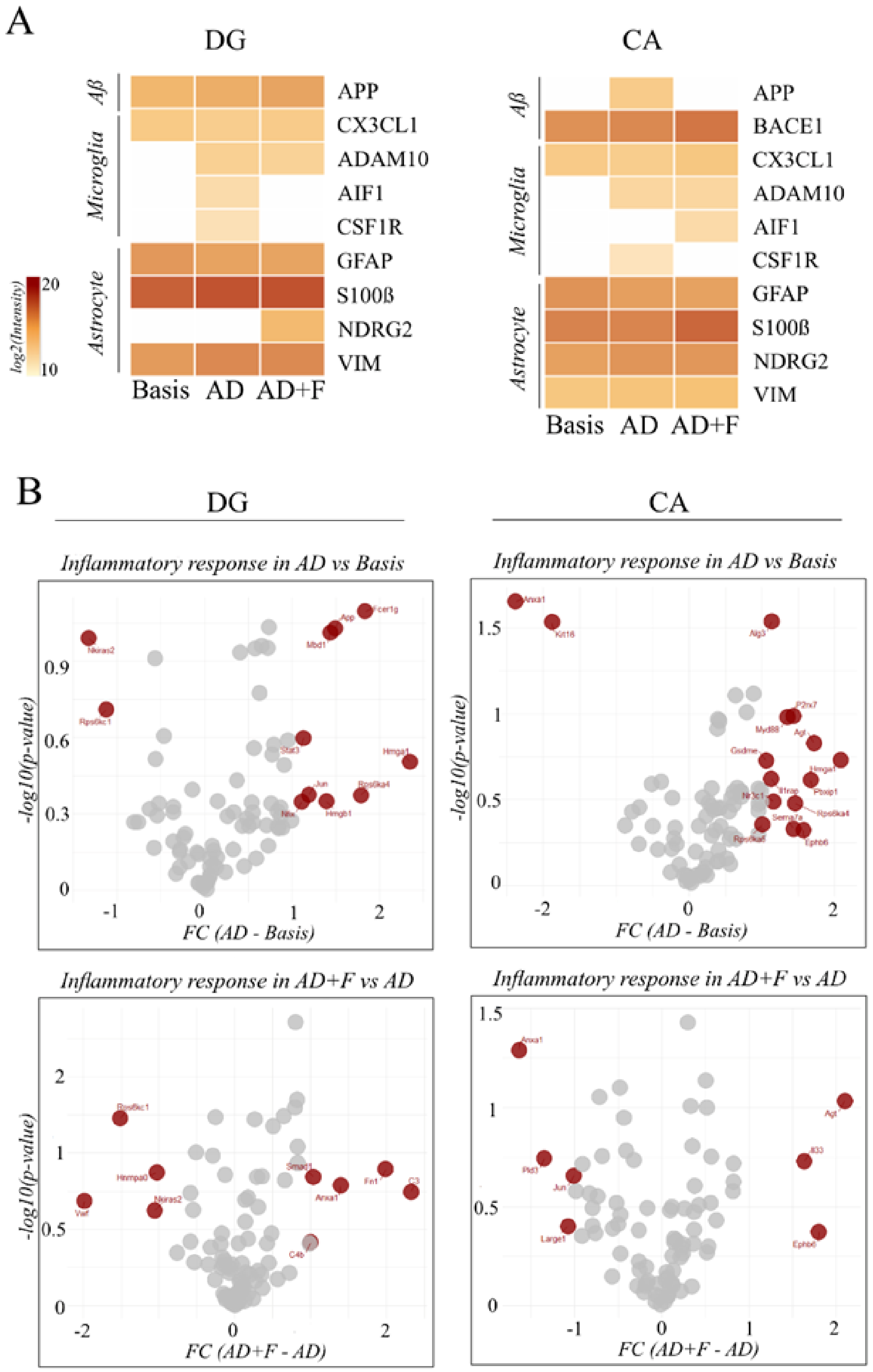


**Supplementary Figure S4.** A) Protein markers for Aβ production, microglia and astrocytes in hippocampal areas DG and CA. Quantification by LCM LC-MS/MS. Intensity values are displayed as log2 across Basis, AD and AD+F groups. B) Enrichment analysis of inflammatory response proteins in AD compared to basis group (left) and AD compared to AD+F (right) in DG and CA. Significantly enriched proteins are indicated in red, non-significant proteins in grey. Statistical test performed by DeqMS. Statistical significance at $p < 0.05$. Enrichment was defined as log2FC > 1.
