# Supplementary material for: Fibre supplementation alters the gastrointestinal microbiome, the microbial metabolites and indicators of neurodegeneration in a mouse model of Alzheimer´s disease
Source: Sci Rep. 2025 Sep 24;15:32705. doi: 10.1038/s41598-025-20986-8 (PMC12460888; doi:10.1038/s41598-025-20986-8)
Supplement: Supplementary file 2 — Supplementary Material 2 [file 41598_2025_20986_MOESM2_ESM.docx]

**Supplementary Table T1.** Quality control data for gas-chromatographic short chain fatty acid analysis based on a 6-point external standard calibration.

| **Analyte** | **Slope** | **SD** | **LOD (mmol/L)** | **LOQ (mmol/L)** |
| --- | --- | --- | --- | --- |
| Acetic acid | 65.5 | 2.87 | 0.145 | 0.438 |
| Propionic acid | 33.5 | 1.48 | 0.146 | 0.442 |
| iso-Butyric acid | 20.3 | 1.21 | 0.197 | 0.596 |
| n-Butyric acid | 21.5 | 0.99 | 0.152 | 0.461 |
| iso-Valeric acid | 16.2 | 0.79 | 0.161 | 0.488 |
| n-Valeric acid | 16.2 | 0.76 | 0.155 | 0.469 |

Abbreviations: LOD, limit of detection; LOQ, limit of quantification; SD, standard deviation.
