# Supplementary material for: Fibre supplementation alters the gastrointestinal microbiome, the microbial metabolites and indicators of neurodegeneration in a mouse model of Alzheimer´s disease
Source: Sci Rep. 2025 Sep 24;15:32705. doi: 10.1038/s41598-025-20986-8 (PMC12460888; doi:10.1038/s41598-025-20986-8)
Supplement: Supplementary file 6 — Supplementary Material 6 [file 41598_2025_20986_MOESM6_ESM.docx]

**Supplementary Table T5.** Concentration of the short-chain fatty acids in caecum and colon content of the three experimental groups.

| **Compound**  *[mmol/L]* | **Basis** | | | | | **AD** | | | | | | | **AD+F** | | | | | | | ***p*** |
| --- | --- | --- | --- | --- | --- | --- | --- | --- | --- | --- | --- | --- | --- | --- | --- | --- | --- | --- | --- | --- |
|  | Mean ± SD | Median | | Range | | Mean ± SD | Median | | | Range | | | Mean ± SD | Median | | | Range | | |  |
| Caecum | | | | | | | | | | | | | | | | | | | | |
| Acetic acid | 39.8^a^ ± 10.4 | | 42.1 | 23.8-52.8 | | 63.4^b^ ± 15.6 | | 61.0 | | | 43.5-96.8 | | 78.7^c^ ± 19.5 | | 75.8 | | | 51.2-113.4 | | < 0.0001 |
| i-Butyric acid | 0.14^a,b^ ± 0.05 | | 0.15 | 0.07-0.21 | | 0.22^a^ ± 0.11 | | 0.21 | | | 0.07-0.52 | | 0.14^b^ ± 0.06 | | 0.13 | | | 0.06-0.25 | | < 0.05 |
| n-Butyric acid | 8.9 ± 2.4 | | 8.9 | 4.7-12.6 | | 13.9 ± 3.7 | | 14.3 | | | 7.4-19.6 | | 14.1 ± 3.7 | | 13.7 | | | 6.6-21.3 | | 0.339 |
| i-Valeric acid | 0.09^a^ ± 0.04 | | 0.11 | 0.03-0.16 | | 0.21^b^ ± 0.15 | | 0.19 | | | 0.05-0.67 | | 0.09^a^ ± 0.05 | | 0.09 | | | 0.02-0.20 | | < 0.0001 |
| n-Valeric acid | 0.22 ± 0.05 | | 0.22 | 0.15-0.32 | | 0.34 ± 0.11 | | 0.34 | | | 0.22-0.55 | | 0.22 ± 0.08 | | 0.21 | | | 0.07-0.37 | | 0.080 |
| Propionic acid | 3.1^a^ ± 1.4 | | 2.7 | 1.5-6.0 | | 7.7^b^ ± 2.3 | | 6.9 | | | 4.4-13.3 | | 8.2^b^ ± 4.9 | | 7.7 | | | 2.5-22.1 | | < 0.01 |
| Colon | | | | | | | | | | | | | | | | | | | | |
| Acetic acid | 26.4^a^ ± 10.5 | | 23.0 | | 14.6-45.9 | 50.4^b^ ± 12.8 | | | 49.3 | | | 27.7-77.2 | 68.4^c^ ± 21.1 | | | 68.5 | | | 38.3-98.9 | < 0.0001 |
| i-Butyric acid | 0.13 ± 0.04 | | 0.13 | | 0.06-0.21 | 0.18 ± 0.09 | | | 0.17 | | | 0.07-0.38 | 0.14 ± 0.08 | | | 0.13 | | | 0.05-0.28 | 0.109 |
| n-Butyric acid | 8.1 ± 4.7 | | 5.9 | | 2.4-15.2 | 8.9 ± 6.3 | | | 7.9 | | | 0.8-26.2 | 16.8 ± 9.9 | | | 13.3 | | | 5.7-36.4 | 0.09 |
| i-Valeric acid | 0.10 ± 0.05 | | 0.10 | | 0.01-0.20 | 0.20 ± 0.14 | | | 0.15 | | | 0.03-0.49 | 0.13 ± 0.07 | | | 0.13 | | | 0.03-0.23 | < 0.05 |
| n-Valeric acid | 0.29 ± 0.18 | | 0.25 | | 0.09-0.69 | 0.38 ± 0.20 | | | 0.36 | | | 0.11-0.92 | 0.41 ± 0.23 | | | 0.25 | | | 0.18-0.73 | 0.738 |
| Propionic acid | 2.1^a^ ± 0.8 | | 1.9 | | 1.3-3.7 | 6.1^b^ ± 1.9 | | | 5.9 | | | 3.4-10.0 | 7.3^b^ ± 4.6 | | | 7.0 | | | 2.6-14.4 | < 0.001 |
| Within one line, means sharing the same superscript letter do not differ significantly (*p* < 0.05); the p-values refer to the comparisons between the three groups within one line each; SD = standard deviation. | | | | | | | | | | | | | | | | | | | | |
